# Pandemic (H1N1) 2009 Cases, Buenos Aires, Argentina

**DOI:** 10.3201/eid1602.091114

**Published:** 2010-02

**Authors:** Marcela Echavarría, Marcia Querci, Débora Marcone, Cristina Videla, Alfredo Martínez, Pablo Bonvehi, Guadalupe Carballal

**Affiliations:** Centro de Educación Médica e Investigaciones Clínicas (CEMIC) University Hospital, Buenos Aires, Argentina

**Keywords:** H1N1, influenza, real-time PCR, influenza-like illness, mortality, hospitalization, pandemic, pandemic (H1N1) 2009, expedited, dispatch

## Abstract

To determine clinical and virologic characteristics of pandemic (H1N1) 2009 in Buenos Aires, Argentina, we conducted real-time reverse transcription–PCR on samples from patients with influenza-like illness, June 11–30, 2009. Of 513 patients tested, 54% were positive for influenza virus subtype H1N1. Infection rate was lowest for patients ≥60 years of age.

A novel influenza A (H1N1) virus emerged in mid-April 2009 and spread rapidly among humans worldwide (*1*,*2*). The virus, pandemic (H1N1) 2009 virus, is a unique combination of gene segments from North American and Eurasian swine lineages and ancestral genes derived from avian species and humans (*3*,*4*). In Buenos Aires, Argentina, the first laboratory-confirmed case of pandemic (H1N1) 2009 was documented May 16, 2009, in a patient who had traveled to the United States. This index case seeded an elementary school outbreak in Buenos Aires, and, within days, several schools reported increasing numbers of cases. The public health response included closure of schools with laboratory-confirmed influenza subtype H1N1 cases, voluntary self-isolation and oseltamivir treatment of suspected and confirmed patients, and recommended chemoprophylaxis for contacts. Despite these interventions, cases increased among the school-age and general population (*5*).

In early June, the number of confirmed cases increased among young adults in Buenos Aires. In addition, cases more severe than those previously seen in school students were being identified among young adults and pregnant women, some of whom required hospitalization in intensive care units. On June 11, 2009, the World Health Organization declared a phase 6 pandemic of this subtype H1N1 virus (*6*).

Argentina’s National Reference Laboratory (Instituto Nacional de Enfermedades Infecciosas, Administración Nacional de Laboratorios e Institutos de Salud, ANLIS, “Dr. Carlos G. Malbrán”) was initially designated as the only approved laboratory for diagnosing pandemic (H1N1) 2009 virus infections. After the rapid increase in the number of cases and the elevation of the pandemic alert level, the virology laboratory at Centro de Educación Médica e Investigaciones Clínicas (CEMIC) University Hospital in Buenos Aires also began diagnosing infections. This report describes clinical and virologic findings for the initial cases of pandemic (H1N1) 2009 diagnosed at CEMIC.

## The Study

Study patients were enrolled from June 11 through 30, 2009, a time that corresponds to the end of autumn and the beginning of winter in the southern hemisphere. Patients seeking care at CEMIC University Hospital and other healthcare providers in Buenos Aires (Federal District) were enrolled if they met the criteria for an influenza-like illness. During the study period, respiratory samples from 513 patients were submitted to CEMIC’s virology laboratory for pandemic (H1N1) 2009 testing.

Combined nasal and pharyngeal swab specimens or nasopharyngeal or tracheal aspirates were obtained from patients and shipped to CEMIC in viral transport media. Epidemiologic and demographic parameters and information on clinical signs, underlying disease, chest radiographs, and medication use were obtained for each patient.

A laboratory diagnosis of influenza infection was determined by real-time reverse transcription–PCR (RT-PCR), by using the Centers for Disease Control and Prevention’s protocol, on a Smart Cycler II (Cepheid, Sunnyvale, CA, USA) (*7*). The QIAamp Viral RNA Mini Kit (QIAGEN, Valencia, CA, USA) was used to extract RNA. Statistical analyses were performed by using χ^2^ and Fisher exact tests.

Of 513 patients, 275 (54%) had positive results for influenza subtype H1N1 by RT-PCR (Table 1). Of these 275 case-patients, 197 (72%) were outpatients and 78 (28%) were hospital inpatients.

**Table 1:** Case-patients with pandemic (H1N1) 2009, by age group, CEMIC University Hospital, Buenos Aires, Argentina, June 11–30, 2009

Case-patient age, y	No. (%) case-patients tested	Total case-patients*				Hospitalized case-patients		
		No. (%)†	95% CI‡	p value		No. (%)§	95% CI‡	p value
<5	63 (12)	37 (59)	45.6–71.0	0.019		19 (51)	34.4–68.0	0.352
5–18	44 (9)	19 (43)	28.3–59.0	0.631		3 (16))	3.4–39.6	0.250
19–59	338 (66)	194 (57)	51.9–62.7	0.003		47 (24)	18.4–30.9	0.304
>60¶	68 (13)	25 (37)	25.4–49.3	—		9 (36)	17.9–57.4	—
Total	513 (100)	275 (54)	49.2–58.0	—		78 (28)	23.1–34.1	—

*Patients with subtype H1N1 infection confirmed by real-time reverse transcription–PCR. †Percent of all patients tested. ‡CI, confidence interval. §Percent of all patients with influenza virus subtype H1N1 infection. ¶Reference group.

Pandemic (H1N1) 2009 was detected most frequently among patients <5 years of age (59%), followed by those 19–59 years of age (57%). The lowest prevalence (37%) was among those >60 years of age; the difference in prevalence among those in this group and those in the groups <5 and 19–59 years of age was significant (p = 0.019 and p = 0.003, respectively) (Table 1). The highest percentage of hospitalizations occurred among patients <5 years of age (51%), followed by those >60 years of age (36%). Among patients 19–59 years of age, 24% required hospitalization (Table 1).

The first case-patient with pandemic (H1N1) 2009 was admitted to CEMIC on June 4, 2009. The patient, a 29-year-old woman in week 36 of pregnancy, had no underlying diseases and required mechanical ventilation within 24 hours of admission and for the next 33 days. During this period, 35 other case-patients with pandemic (H1N1) 2009 were hospitalized at CEMIC; of these 36 patients, 16 (44%) were admitted to the intensive care unit, and 10 (28%) required mechanical ventilation. The most common diagnosis for hospitalized patients was pneumonia; 42% had interstitial bilateral infiltrates, 31% had focal consolidation, and 4% had pleural effusion. Chest radiographs were unremarkable for 23% of hospitalized patients. The mean hospital stay was 7 days (range 2–42 days; median 9 days).

A total of 78 case-patients were hospitalized at CEMIC and other institutions in Buenos Aires. Six patients died, of whom 5 had an underlying condition: 3 were immunocompromised, 1 was obese and a chronic user of tobacco, and 1 had pulmonary and heart disease. Three patients were >60 years of age, 2 were in the 19–59-year age group, and 1 was an infant. Two hospitalized patients died at CEMIC. Both were >60 years of age, had underlying health conditions, and later developed bacterial sepsis; 1 also had bilateral pneumonia.

Underlying conditions were evaluated for all 275 patients with laboratory-confirmed influenza virus subtype H1N1 infection. For evaluation, hospitalized and ambulatory patients were divided into 2 age groups: <5 and >5 years of age. For patients >5 years of age, the presence of an underlying condition was significantly associated with a higher rate of hospitalization (p = 0.040). Of the 78 hospitalized patients, 56 (72%) had no underlying condition (Table 2).

**Table 2:** Underlying clinical conditions in 275 case-patients with pandemic (H1N1) 2009, by age group, CEMIC University Hospital, Buenos Aires, Argentina, June 11–30, 2009

Clinical condition	Case-patients <5 y of age				Case-patients >5 y of age		
	No. (%) hospitalized	No. (%) outpatients	p value		No. (%) hospitalized	No. (%) outpatients	p value
None*	15 (79)	16 (89)	—		41 (69)	148 (83)	—
Underlying condition	4 (21)	2 (11)	0.659		18 (31)	31 (17)	0.040
Immunocompromised	1 (5)	1 (5.5)	0.999		9 (15)	11 (6)	0.028
Pregnant	0	0	—		1 (2)	4 (2)	0.999
Other†	3 (16)	1 (5.5)	0.603		8 (14)	16 (9)	0.205
Total	19	18	—		59	179	—

*Reference group. †Hematologic malignancies, chronic lung disease (asthma, chronic obstructive pulmonary disease), chronic cardiac disease, diabetes and obesity.

## Conclusions

From June 11–30, 2009, pandemic (H1N1) 2009 virus was detected in 54% of respiratory samples submitted to CEMIC’s virology laboratory. This percentage is similar to the overall percentage reported by Argentina’s National Reference Laboratory during the same period (*8*). However, initial reports from Mexico showed a lower positivity rate (29%, 2,582/8,817) (*9*). This difference may be partly caused by the different clinical criteria for respiratory specimen collection, site restrictions, or seasonal factors. The percentage of influenza subtype H1N1 cases detected during this study (54%) is apparently higher than that for seasonal influenza A strains (H1N1 and H3N2) detected at CEMIC University Hospital during the same epidemiologic weeks in previous years (19% and 26% in children and adults, respectively, as confirmed by immunofluorescence testing) (*10*) (M. Echavarría, unpub. data).

The high frequency of infection with pandemic (H1N1) 2009 virus likely resulted from the lack of population immunity to this antigenically novel H1N1 subtype. The lowest rate of infection was seen for patients >60 years of age. This finding may suggest that persons in this age group were previously exposed, through infection or vaccination, to an influenza A (H1N1) virus that is genetically and antigenically more closely related to pandemic (H1N1) 2009 than to other recent influenza A viruses (*11*).

Our finding that 66% of respiratory samples tested during this study were from patients 19–59 years of age is similar to findings in previous pandemic influenza situations. Furthermore, 49% of hospitalized patients in this study were 19–59 years of age. Most case-patients hospitalized with pandemic (H1N1) 2009 infection (72%) had no underlying medical condition, but a severe degree of lung involvement was observed in these patients: one third required mechanical ventilation support.

We report a mortality rate of 2% among the 275 confirmed pandemic (H1N1) 2009 case-patients followed in this study. A similar rate (2.3%) was reported by the National Ministry of Health during the same period (*8*). From May through December 4, 2009, a total of 11,234 cases were confirmed in Argentina, and 613 (5.5%) case-patients died (*5*). In the Americas, Argentina currently has the fourth highest number of deaths associated with influenza virus subtype H1N1, after the United States, Brazil, and Mexico (*12*). The reasons for these unusual epidemiologic features are the focus of ongoing investigations.
